# Transcriptome reprogramming through alternative splicing triggered by apigenin drives cell death in triple-negative breast cancer

**DOI:** 10.1038/s41419-023-06342-6

**Published:** 2023-12-13

**Authors:** Meenakshi Sudhakaran, Tatiana García Navarrete, Katherine Mejía-Guerra, Eric Mukundi, Timothy D. Eubank, Erich Grotewold, Daniel Arango, Andrea I. Doseff

**Affiliations:** 1 Molecular, Cellular, and Integrative Physiology Graduate Program, Michigan State University, East Lansing, MI USA; 2 Department of Biochemistry and Molecular Biology, Michigan State University, East Lansing, MI USA; 3 Department of Molecular Genetics, The Ohio State University, Columbus, OH USA; 4 Department of Microbiology, Immunology & Cell Biology, West Virginia University, Morgantown, WV USA; 5 Department of Pharmacology and Robert H. Lurie Comprehensive Cancer Center, Feinberg School of Medicine, Northwestern University, Chicago, IL USA; 6 Department of Physiology and Department of Pharmacology and Toxicology, Michigan State University, East Lansing, MI USA

**Keywords:** Cancer, Transcriptomics

## Abstract

Triple-negative breast cancer (TNBC) is characterized by its aggressiveness and resistance to cancer-specific transcriptome alterations. Alternative splicing (AS) is a major contributor to the diversification of cancer-specific transcriptomes. The TNBC transcriptome landscape is characterized by aberrantly spliced isoforms that promote tumor growth and resistance, underscoring the need to identify approaches that reprogram AS circuitry towards transcriptomes, favoring a delay in tumorigenesis or responsiveness to therapy. We have previously shown that flavonoid apigenin is associated with splicing factors, including heterogeneous nuclear ribonucleoprotein A2 (hnRNPA2). Here, we showed that apigenin reprograms TNBC-associated AS transcriptome-wide. The AS events affected by apigenin were statistically enriched in hnRNPA2 substrates. Comparative transcriptomic analyses of human TNBC tumors and non-tumor tissues showed that apigenin can switch cancer-associated alternative spliced isoforms (ASI) to those found in non-tumor tissues. Apigenin preferentially affects the splicing of anti-apoptotic and proliferation factors, which are uniquely observed in cancer cells, but not in non-tumor cells. Apigenin switches cancer-associated aberrant ASI in vivo in TNBC xenograft mice by diminishing proliferation and increasing pro-apoptotic ASI. In accordance with these findings, apigenin increased apoptosis and reduced tumor proliferation, thereby halting TNBC growth in vivo. Our results revealed that apigenin reprograms transcriptome-wide TNBC-specific AS, thereby inducing apoptosis and hindering tumor growth. These findings underscore the impactful effects of nutraceuticals in altering cancer transcriptomes, offering new options to influence outcomes in TNBC treatments.

## Introduction

Triple-negative breast cancer (TNBC) is a highly aggressive metastatic tumor characterized by a lack of estrogen, progesterone, and HER2 receptors, accounting for approximately 20% of all breast cancers (BC) [1]. TNBC has a poor clinical prognosis due to the lack of targeted therapies and refractory behavior, underscoring the need to identify new alternative therapeutic approaches.

TNBC cells exhibit remarkable transcriptome alterations, including dysregulation of alternative splicing (AS) [2, 3]. Aberrant cancer-specific alternative spliced isoforms (ASI) promote tumor development, metastasis, and resistance to anti-tumor therapies [4]. Thus, identifying novel approaches that can modulate TNBC transcriptomes is warranted to prevent tumor development and improve therapeutic outcomes.

Flavonoids are the largest class of plant-specialized metabolites with health benefits that constitute key dietary nutraceuticals. Apigenin (4′,5′,7-trihydroxyflavone), a flavonoid abundant in celery and parsley, has shown anti-carcinogenic activity in different model systems, including BC [5–8]. We demonstrated that apigenin sensitizes TNBC spheroids and patient-derived TNBC xenografts (PDX) to doxorubicin-induced apoptosis [7]. Moreover, apigenin sensitizes primary patient-derived lung cancer cells to TNF-related apoptosis-inducing ligand (TRAIL) anti-tumor therapies, without affecting non-tumor cells [9]. Screening of a human BC phage display library coupled with next-generation sequencing (PD-Seq) identified direct targets of apigenin [10]. Among the high-affinity proteins that bind to apigenin, we found RNA binding proteins (RBPs), Heterogeneous Nuclear Ribonucleoprotein A2/B1 (hnRNPA2), Musashi 2 (MSI2), CUG-BP ELav-Like Family Member 1 (CELF1) [10], which are key regulators of AS. In TNBC cells, apigenin alters the AS of caspase-9 and cellular FLICE-inhibitory protein *(*c-FLIP, encoded by the *CFLAR* gene), a regulator of caspase-8-dependent apoptosis [10]. Apigenin altered the AS of *CFLAR* and TRAIL death receptor 5 (*DR5*), resulting in the sensitization of human primary tumor-derived lung cancer cells to TRAIL [9]. However, whether apigenin affects the aberrant AS landscape associated with TNBC remains unknown.

Here, we conducted comprehensive transcriptomic analyses to study the effects of apigenin on AS in human TNBC MDA-MB-231 cells. Our study provides mechanistic insights into the anti-cancer effects of apigenin. Most AS events altered by apigenin were enriched in hnRNPA2 substrates compared to those of MSI2 or CELF1. The genes affected by apigenin through AS were enriched in cell death. Comparative analyses using available human transcriptome datasets showed that apigenin can modulate aberrant ASI commonly found in the tumors of patients with TNBC. Moreover, apigenin switched the AS profiles of TNBC cells by increasing the expression of molecules that either induced apoptosis or inhibited cell proliferation. Importantly, these changes in AS were also observed in vivo in mammary tumor tissues. Consistently, apigenin decreased the proliferation and induced apoptosis of TNBC cells in a xenograft mouse model. Our findings reveal the impact of apigenin on the TNBC transcriptome, providing novel mechanistic insights into how this dietary nutraceutical confers anti-cancer activity through splicing reprogramming. These findings underscore the potential of nutraceuticals in improving TNBC prevention and treatment.

## Material and methods

The data generated in this study are available in the article and its supplementary files.

### Cell culture

Human TNBC MDA-MB-231 cells (Cat. #: HTB-26™, RRID: CVCL_0062), BT-549 cells (Cat#: HTB-122™, RRID:CVCL_1092), MDA-MB-468 cells (Cat#: HTB-132™, RRID:CVCL_0062) and immortalized breast epithelial MCF10A cells (Cat. #: CRL-10317™, RRID: CVCL_0598) were purchased from the American Type Culture Collection (ATCC). MDA-MB-231 cells were cultured in Dulbecco’s Modified Eagle’s medium (DMEM) supplemented with 5% Fetal Bovine Serum (FBS) and 1% penicillin/streptomycin (P/S) antibiotics. MCF10A cells were grown in DMEM/F12 supplemented with 10% FBS, 1% P/S, and 10 ng/ml Epidermal Growth Factor (ProteinTech, Cat. #: HZ-1326), 0.5 mg/ml hydrocortisone (Sigma-Aldrich, Cat. # H4001, 100 ng/ml cholera toxin (Sigma-Aldrich, Cat. #: C8052), and 10 μg/ml insulin (Sigma-Aldrich, Cat. #: I3536). All cell lines were treated with 50 μM apigenin (Sigma-Aldrich, Cat. # A3145), or DMSO (Sigma-Aldrich, Cat.#: D2650) for 48 h in all experiments.

### Animal studies

All procedures were approved by The Ohio State University Institutional Animal Care and Use Committee (IACUC, protocol A0208). Mice were housed under constant humidity (50 ± 5%), temperature (22 ± 2 °C), and a 12-h day/night cycle and received food and water *ad libitum*. Severe combined immuno-deficient 6–10-week-old female mice were injected with 10^6^ MDA-MB-231 cells into the mammary fat pad. Mice were injected daily intraperitoneally with 25 mg/kg apigenin dissolved in 100 μl vehicle [20% DMSO, 10% ethanol, 30% KolliphorEL (Sigma-Aldrich, Cat. #:C5135), and 40% PBS], or vehicle alone for 28 days. Tumor volume was measured three times per week using calipers (tumor volume = *width* * *lenght*^2^/2). Mice were euthanized on day 28, and tumors were removed, measured, and snap-frozen in liquid nitrogen or fixed in 10% buffered formalin.

### RNA-seq analyses

Library preparation and sequencing were performed by BGI (https://www.bgi.com/us). Briefly, indexed libraries from isolated RNA were generated for three independent biological replicates using the DNBseq strand-specific transcriptome library kit following the manufacturer’s instructions. Libraries were validated on an Agilent Bioanalyzer 2100 and sequenced using paired-end 100-mer reads in the DNBSEQ-G400 sequencer, yielding an average of 40 million reads per sample. Quality and duplication levels were evaluated by FastQC (V.0.11.8) and aligned to the human genome assembly 19 (hg19) using TopHat (V.1.4.1) with default parameters [11]. The annotations for all human gene models were obtained from RefSeq hosted at UCSC. Uniquely aligned reads were kept for further analysis. Raw read counts were obtained using HTseq (V.0.11.0) [12], loaded into R (V.4.3.1), and normalized for library sizes using DESeq2 [13]. Replicate reproducibility was evaluated by hierarchical clustering (complete linkage method) using the Euclidean distances between the samples and by principal component analysis using DESeq2.

### AS analyses

To detect differential AS events, we used the Mixture of Isoforms (MISO) package (version 0.5.4) with the default parameters [14]. First, the coordinates for the exon-centric events for the major classes of mRNA processing events and the isoform-centric annotations were retrieved using the annotation files for the hg19 version. The Percentage of Spliced Index (PSI) and the difference in PSI (ΔPSI) between samples treated with apigenin or DMSO were computed for all events in each of the three biological replicates. Because MISO does not accept replicates, we pooled the biological replicates and computed all events in the pooled dataset. Events with a Bayes factor ≥ 20 in the pooled dataset showing the same ΔPSI direction in each biological replicate were selected as differentially affected events. AS events were visualized using the Integrated Genome Viewer (IGV; V.2.13.2) [15] and represented as Sashimi plots. Downstream data analyses were performed using R.

Publicly available RNA-seq datasets from normal breast tissue (NBT) and TNBC patient samples were obtained from the Gene Expression Omnibus repository (GEO: GSE52194, GSE142731, GSE58135), and changes in AS were analyzed using the same workflow described above.

### Statistical tests

The sample sizes and statistical tests for each plot are specified in each figure legend. For all graphs, *p* ≤ 0.05 is significant.

## Results

### Apigenin induces apoptosis and modulates AS transcriptome wide in human TNBC cells

**Fig. 1:**
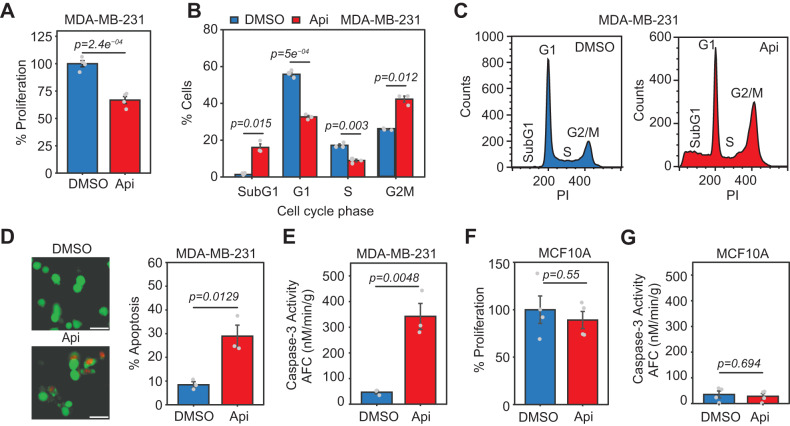
Apigenin has anti-carcinogenic activity in TNBC cells with no effect in non-tumor mammary epithelial cells. MDA-MB-231 cells were treated with 50 μM apigenin (Api) or DMSO for 48 h. **A** Cell proliferation was assessed using MTS assays. **B** Bar plots represent the percent of cells in each phase of the cell cycle. **C** Representative cell cycle profiles in cells stained with propidium iodide (PI). **D** Percentage of apoptotic cells evaluated by fluorescence microscopy of cells stained with calcein AM/PI (scale bar indicates 100 μm). **E** Caspase-3 activity in MDA-MB-231 cells. **F**, **G** MCF10A cells were treated with 50 μM apigenin or DMSO for 48 h. All data represent mean ± SEM, *n* = 3–4. Statistical significance was evaluated by two-tail *t*-test for **A** and **D**–**G** and pairwise *t*-test for **B**.

**Fig. 2:**
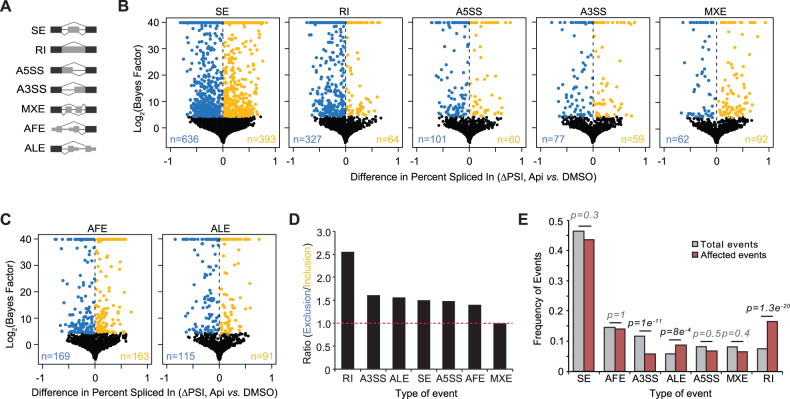
Apigenin alters transcriptome-wide AS in TNBC cells. RNA-seq analyses were performed in MDA-MB-231 cells treated with 50 μM apigenin (Api) or DMSO for 48 h. **A** Schematic representation of all types of mRNA processing events. **B**, **C** Volcano plots depicting changes in the percentage of spliced isoform index (ΔPSI) in apigenin- *vs*. DMSO-treated cells. Blue and yellow dots represent significantly excluded and included AS events, respectively. **D** Ratio of the excluded *vs*. included AS events. A ratio higher than 1 implies that the number of excluded AS events is higher in cells treated with apigenin. **E** The proportion of differentially spliced events by apigenin compared to the total number in each category. Statistical significance was evaluated by proportion with Bonferroni correction.

To evaluate the effect of apigenin on TNBC transcriptomes, RNA-seq was performed using mRNA from MDA-MB-231 TNBC cells treated with 50 μM apigenin or diluent DMSO for 48 h, a concentration and time that significantly decreased cell proliferation, induced cell cycle arrest, and promoted apoptosis in cancer cells (Fig. 1A–E), without affecting the proliferation or apoptosis of non-tumor MCF10A cells (Fig. 1F, G). Hierarchical clustering and normalized read count correlations showed high concordance between replicates and significant differences between the transcriptome profiles of cells treated with apigenin or the diluent control (Supplementary Fig. 1A–C). We next analyzed the effect of apigenin on the expression of different mRNA processing events using the exon-centric Mixture of Isoforms (MISO) package [14]. We examined the skipped exon (SE), retained intron (RI), alternative 5′ splice site (A5SS), alternative 3′ splice site (A3SS), and mutually exclusive exon (MXE). In addition, we measured alternative first exon (AFE), and alternative last exons (ALE), which are generated through alternative transcription initiation and alternative 3′ mRNA cleavage rather than AS [16, 17] (Fig. 2A). Of the 80,501 mRNA processing events examined, apigenin significantly affected 2409 (Fig. 2B and Supplementary Table 1). Overall, we found that apigenin induced a more significant proportion of exon or intron exclusions, as illustrated by a higher number of events with a negative ΔPSI (blue) compared to those with a positive ΔPSI (yellow, Fig. 2B, C and Supplementary Fig. 2A), which was most evident in RI events (327 blue *vs*. 64 yellow, Fig. 2D). Three types of mRNA processing patterns, RI, A3SS, and ALE, were significantly affected by apigenin (Fig. 2E, brown vs. gray). We found that RI and ALE were enriched in the presence of apigenin, while the frequency of A3SS decreased. We next used MISO to identify changes in the expression of whole transcript isoforms through the isoform-centric analysis [14]. We found that apigenin altered 5362 mRNA isoforms in 3300 genes (Supplementary Fig. 2B and Supplementary Table 2), confirming the transcriptome-wide effects of apigenin on mRNA processing. To confirm the validity of several AS events identified by RNA-seq analyses, we used isoform-specific primers and RT-PCR analyses (Supplementary Fig. 2C and Supplementary Table 3). The analyses of additional AS events confirmed good concordance between the RT-PCR and RNA-seq results (Supplementary Fig. 2C, D, *R*^2^ = 0.92). Taken together, these results show that apigenin reprograms transcriptome-wide mRNA processing.

### Alternative splicing of hnRNPA2 substrates is significantly affected by apigenin

**Fig. 3:**
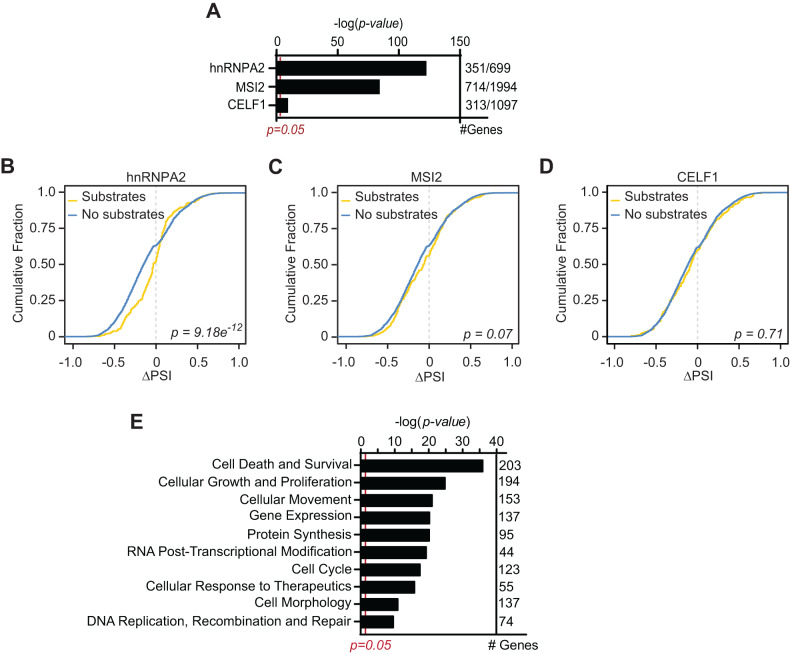
Apigenin regulates hnRNPA2 substrates. **A** The enrichment of hnRNPA2, MSI2, and CELF1 substrates among the genes differentially spliced in MDA-MB-231 cells treated with 50 μM apigenin. Enrichment was evaluated by a hypergeometric distribution test. **B**–**D** The genes differentially spliced in cells treated with apigenin were segregated as hnRNPA2 (**B**), CELF1 (**C**), or MSI2 (**D**) substrates (yellow) or non-substrates (blue) to compare the cumulative fractions of ΔPSI. **E** Molecular functional enrichment of hnRNPA2 substrates altered by apigenin through AS using IPA. Statistical significance was evaluated by Kolmogorov–Smirnov (K–S) test for **B**–**D** and by Fisher’s exact test for **E**.

We have previously shown that apigenin associates with hnRNPA2, MSI2, and CELF1 [9, 10]. Thus, we hypothesized that apigenin affects the AS of the substrates of such RBPs. To investigate this possibility, we intersected the genes whose AS was affected by apigenin with experimentally validated substrates of hnRNPA2, MSI2, or CELF1 identified by CLIP-seq (Crosslinking and Immunoprecipitation followed by deep sequencing) in cancer cells (Supplementary Table 4) [18–20]. We found that apigenin significantly affected the AS of genes encoding hnRNPA2, MSI2 and CELF1 substrates (Fig. 3A and Supplementary Table 4). Next, we compared the ΔPSI between apigenin and DMSO for each potential splice event in genes that are either substrates or non-substrates of these RBPs. We found that the ΔPSI magnitude was significantly different among genes that are substrates of hnRNPA2 (yellow) compared to those that are not substrates of hnRNPA2 (blue; Fig. 3B). In contrast, no significant difference was observed between substrates and non-substrates of MSI2 or CELF1 (Fig. 3C, D), suggesting that the effect of apigenin is preferentially mediated through hnRNPA2 targets. Pathway analyses of the 351 genes (Fig. 3A), which are both affected by apigenin through AS and are hnRNPA2 substrates, showed enrichment in cell death and survival (Fig. 3E). Taken together, these results suggest that the physiological effect of apigenin in TNBC is mainly mediated by its impact on the splicing of hnRNPA2 substrates that are involved in cell death and survival.

### AS events affected by apigenin regulate cell death and survival in TNBC cells

**Fig. 4:**
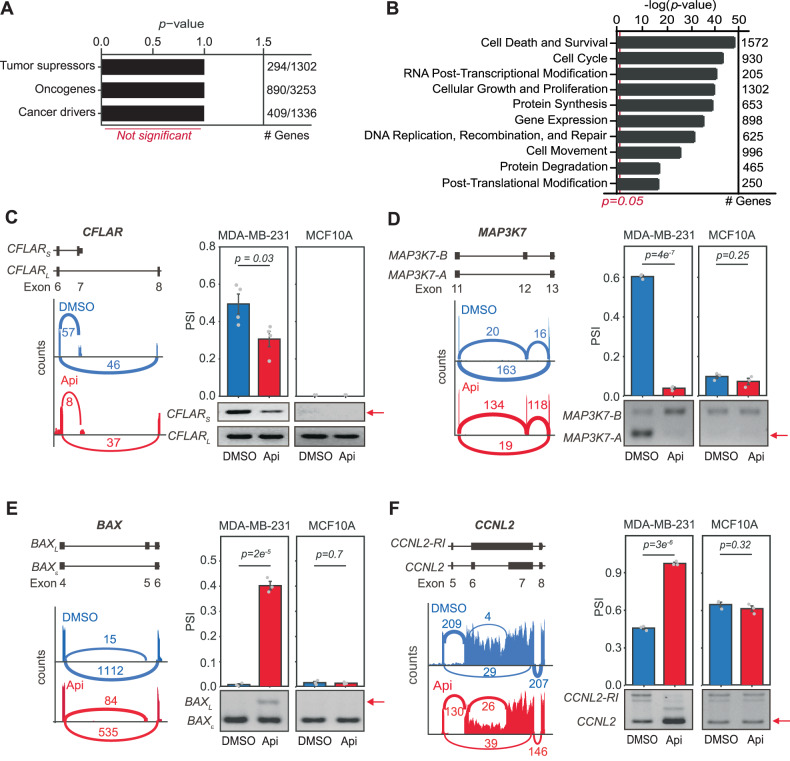
Apigenin modulates the splicing of genes involved in cell proliferation and apoptosis in TNBC cells. **A** Enrichment analysis of tumor suppressors, oncogenes, and cancer drivers among the genes differentially spliced in cells treated with apigenin vs. DMSO was evaluated by a hypergeometric test. **B** Molecular functional enrichment analyses using IPA of genes differentially spliced in cells treated with apigenin. Statistical significance was evaluated by Fisher’s exact test. **C**–**F** Representative Sashimi plots (left) and PCR (right) validation of AS events in cell death-related genes in MDA-MB-231 and MCF10A cells treated with apigenin (Api) or DMSO control. A representative gel is shown at the bottom. Mean ± SEM of the PSI of the isoform indicated by the red arrow, *n* = 3. Statistical significance was evaluated by two-tail *t*-test.

To evaluate whether apigenin specifically affects the AS of cancer-related genes, we performed gene enrichment analyses focused on the categories of cancer driver genes, oncogenes, and tumor suppressors using 3761 genes, corresponding to 2409 exon-centric (Fig. 2B, C and Supplementary Table 1) and 5363 isoform-centric (Supplementary Fig. 2B and Supplementary Table 2) events affected by apigenin through AS (Supplementary Table 5) [21–24]. We found that AS events affected by apigenin were not enriched among cancer drivers, oncogenes, or tumor suppressors (Fig. 4A, *p* = 1 in all categories). Next, to investigate the biological impact of the apigenin-induced transcriptomic alterations, we conducted functional enrichment analyses of all the 3761 genes affected by apigenin through AS. The changes in AS induced by apigenin were significantly enriched in genes that regulate cell death and survival comprising 1572 genes (Fig. 4B and Supplementary Table 6).

Next, we performed isoform-specific RT-PCR to validate the apigenin-induced changes in several AS events among the 1,572 genes involved in cell death and survival groups representing varying ΔPSI values. *CFLAR* displays reduced read coverage in the alternative last exon 7, specific to *CFLAR* short isoform (*CFLAR*_*S*_), compared to diluent control (Fig. 4C). Consistently, reduced levels of *CFLAR*_*S*_ were observed by isoform-specific PCR in apigenin-treated cells (Fig. 4C). *CFLAR*_*S*_ encodes the cFLIP-S protein, which enhances the inhibition of death receptors compared to the long isoform *CFLAR*_*L*_ [25]. Apigenin affected the splicing of *MAK3K7*, a gene encoding the TAK1 protein. *MAP3K7* has several AS isoforms, including *MAP3K7-B* and *MAP3K7-A* [26]. *MAP3K7-A* lacks exon 12 and enhances cell proliferation through NF-κB signaling in cancer cells, including MDA-MB-231 cells, while the long isoform *MAP3K7-B* promotes apoptosis [27]. Our results showed that apigenin reduced *MAP3K7-A* and increased *MAP3K7-B* expression (Fig. 4D), thereby leading to apoptosis. Apigenin also altered BAX splicing (Fig. 4E) [26], a pro-apoptotic molecule [28]. MDA-MB-231 cells lack *BAX*_*L*_, but express high levels of the shorter isoform *BAXε*. *BAXε* is a result of exon inclusion and frameshift that translates into a protein lacking the BH2 and transmembrane domains required for efficient BAX pro-apoptotic activity [28]. Apigenin significantly increased the levels of *BAX*_*L*_ mRNA, which has enhanced pro-apoptotic activity, compared to *BAXε* (Fig. 4E) [28]. Analysis of *CCLN2*, a splicing regulator that represses cell cycle progression [29], revealed that apigenin reduced *CCLN2-RI*, an isoform that retains intron six resulting in a premature stop codon that translates into a truncated protein (Fig. 4F) [26]. Apigenin increased the expression of translation-competent CCLN2 isoforms that are associated with the inhibition of tumor growth (Fig. 4F) [29].

Next, we compared the AS events mentioned above in human non-tumor breast epithelial MCF10A cells treated with apigenin or a diluent. Our results showed that apigenin did not affect AS in MCF10A cells (Fig. 4C–F, *right*). Further, we investigated if the changes in AS induced by apigenin in MDA-MB-231 cells are extended to other TNBC subtypes. All the apigenin-induced events tested were identical between mesenchymal subtypes MDA-MB-231 and BT-549 cell lines as evaluated by isoform-specific RT-PCR (Supplementary Fig. S3). Only one out the eight apigenin-induced AS events tested (Supplementary Fig. S3; *BAX*) was different in the basal-like MDA-MB-468 cell lines, corresponding to ~88% similarity with mesenchymal TNBC. These findings show that apigenin favors mRNA isoforms, resulting in proteins that increase cell death in different TNBC subtypes without affecting the AS of non-tumor cells.

### Apigenin modulates dysregulated alternative spliced isoforms in TNBC patients

**Fig. 5:**
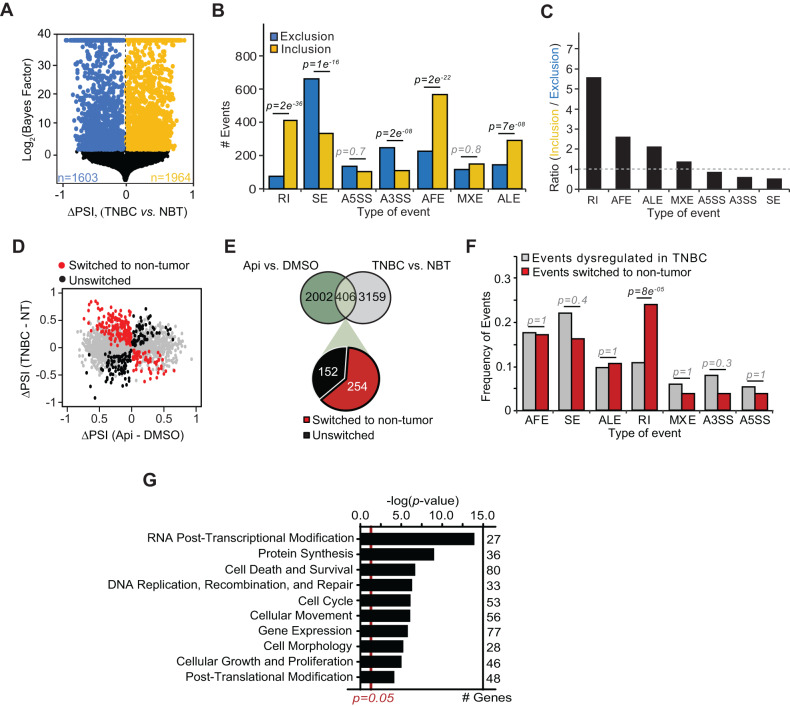
Apigenin restores the splicing of genes dysregulated in TNBC. **A** Volcano plots depict ΔPSI in TNBC vs. NBT mammary tissues. Blue and yellow dots represent AS excluded and included, respectively, in TNBC vs. NBT. **B** The ASIs were segregated as excluded (blue) or included (yellow) in TNBC as compared to NBT. Statistical significance was evaluated by *χ*^2^ test with a Bonferroni correction. **C** Ratio of the number of included/excluded ASIs in TNBC *vs*. NBT. A ratio higher than 1 implies that the number of included ASI is higher in TNBC. **D** Scatter plot of ΔPSI of events dysregulated in TNBC compared to the ΔPSI in apigenin (Api)-treated MDA-MB-231 cells. Red dots indicate events that are switched by apigenin towards non-tumor levels while black dots indicate the non-switched events. **E** Venn diagram intersection of AS events affected by apigenin (Api) in MDA-MB-231 cells and the events dysregulated in TNBC. Among the shared genes, those switched by apigenin towards non-tumor levels are indicated in red. **F** Proportion of ASIs in TNBC compared to the proportion of events switched by apigenin towards non-tumor levels. Statistical significance was evaluated by test of proportion with Bonferroni correction. **G** Molecular functional enrichment analyses using IPA of genes switched by apigenin to non-tumor levels. Statistical significance was evaluated by Fisher’s exact test.

The ability of apigenin to restore dysregulated AS events found in human TNBC MDA-MB-231 cells prompted us to examine whether this effect could also be observed in the transcriptomes of TNBC patients. For this purpose, we first analyzed publicly available RNA-seq data from TNBC and non-tumor breast tissues (NBT) from twenty-six and ten subjects, respectively (data available in GSE52194, GSE58135 and GSE142731) [30–32] using MISO. We found 3567 AS events comprising 2060 genes that were differentially spliced in TNBC compared with NBT (Fig. 5A), suggesting transcriptome dysregulation. Our analyses showed that TNBC was characterized by a higher number of inclusions (yellow) vs. exclusion events (blue) compared to NBT (Fig. 5B). We found that the highest inclusion-to-exclusion ratio was observed in RI events in TNBC (Fig. 5C), which is in agreement with previous reports [30, 33, 34].

We next interrogated whether apigenin changes the ASI found in the transcriptome of TNBC patients. Hence, we intersected the AS events affected by apigenin in MDA-MB-231 cells with those dysregulated in TNBC tumors (Fig. 5D). We found that apigenin altered splicing in 406 TNBC-specific events, corresponding to ~17% of all events affected by apigenin (Fig. 5E), and represented a significant enrichment compared to what would be expected by chance (*p* < 2.2e^−16^). Further analyses indicated that the majority of the changes in AS events, representing ~63% (254 of 406 total affected by apigenin), corresponded to changes from AS events characteristic of TNBC to those found in non-tumor cells (NBT, Fig. 5E). The ASI changed by apigenin from TNBC to NBT profiles showed significant enrichment in RI (Fig. 5F, red vs. gray), whereas all other types of events showed no significant enrichment compared to what was expected by chance (Fig. 5F, red vs. gray). Functional enrichment analyses of all the events switched by apigenin to non-tumor profiles (254 events) revealed RNA-post transcriptional modification was the most significantly enriched group with the highest number of genes affected corresponding to cell death and survival (Fig. 5G). Together, these findings suggest that apigenin reprograms the ASI found in TNBC patients through perturbations in RNA and cell death pathways, inducing a non-tumor-like transcriptome.

### Apigenin alters the AS of genes involved in cell death in vivo decreasing tumor growth

**Fig. 6:**
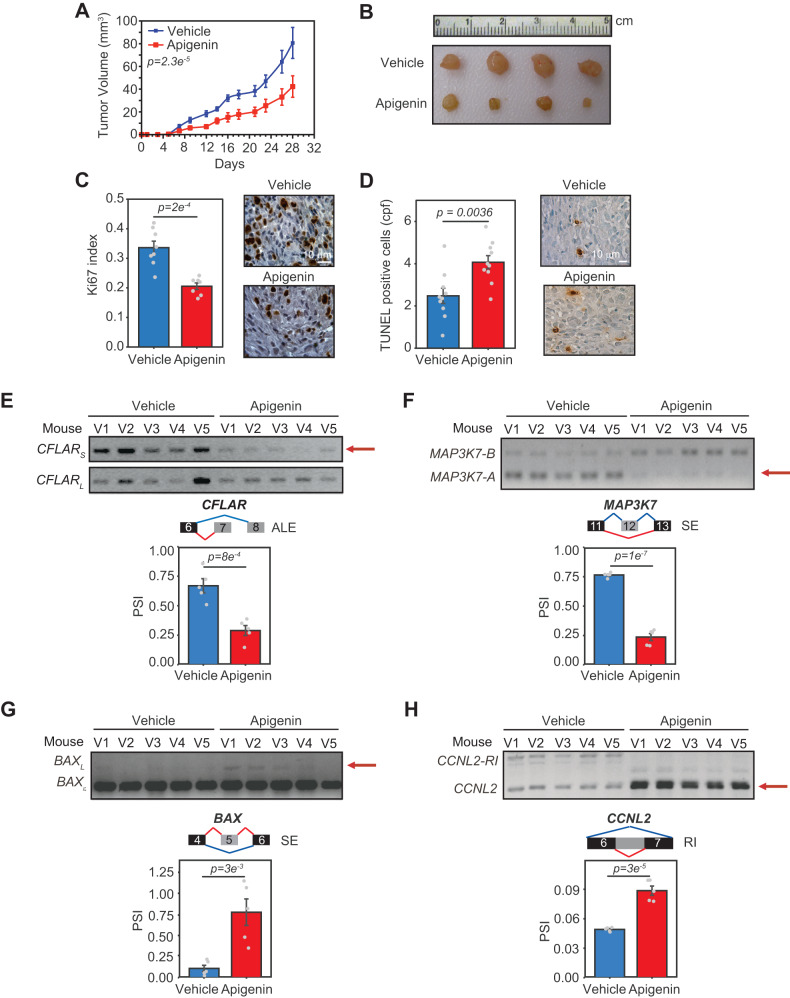
Apigenin decreases tumor growth in vivo altering AS of genes involved in tumorigenesis. **A** Line plots represent the mammary tumor volume in mice bearing MDA-MB-231 xenografts treated with 25 mg/kg apigenin or vehicle for 28 days. Mean ± SEM, *n* = 12. Statistical significance was evaluated by two-way ANOVA. **B** Representative tumors at day 28. **C** Tumor sections were stained by immunohistochemistry using anti-Ki67 antibodies. Ki67 index was calculated using the formula Ki67-positive cells/Total cells. **D** Tumor sections were stained by TUNEL. TUNEL-positive cells were counted and represented as counts per field (cpf). Mean ± SEM, *n* = 12 and statistical significance was evaluated by two-tailed *t*-test (**C**, **D**). **E**–**H** Total RNA was isolated from tumors and the AS of key genes involved in cell death was evaluated by isoform-specific PCR and agarose gel electrophoresis. Bar plots represent the Mean ± SEM of the PSI of the isoform indicated in red. Mean ± SEM, *n* = 5. Statistical significance was evaluated by two-tailed *t*-test.

To gain insight into the functional ability of apigenin to modulate AS in vivo, we evaluated the effect of apigenin on tumor growth. We found that apigenin significantly decreased tumor growth in MDA-MB-231-derived xenografts (Fig. 6A, B), which is in agreement with previous studies [6, 35, 36]. Reduction in tumor size was a consequence of decreased proliferation and increased apoptosis of tumor cells, as determined by in situ immunostaining of tumor sections with the proliferation marker Ki67 and the apoptotic assay Terminal Uridine Nick-End Labeling (TUNEL), respectively (Fig. 6C, D).

To evaluate whether apigenin affected dysregulated TNBC-induced AS events in vivo, we compared the AS patterns of *CFLAR, MAP3K7, BAX,* and *CCNL2* in MDA-MB-231-derived xenografts treated with apigenin or the vehicle control. We found that apigenin significantly reduced pro-survival ASI *CFLAR*_*S*_ and *MAP3K7-A* (Fig. 6E, F). In addition, apigenin increased pro-apoptotic ASI *BAX*_*L*_ and *CCNL2* (Fig. 6G, H). These results show that apigenin can switch the ASI of genes involved in cell death programs in vivo underscoring the anti-carcinogenic mechanisms of this flavonoid.

## Discussion

The high aggressiveness and resistance of TNBC contribute to poor clinical outcomes. TNBC transcriptome landscapes have a myriad of AS changes, characterized by an increase in aberrant TNBC-associated ASI that favors proteins involved in promoting tumor growth and resistance to cell death [30]. These observations underscore the possibility of using approaches that reprogram AS circuits to improve TNBC outcomes. The use of natural compounds, including flavonoids, to induce cancer cell apoptosis or increase sensitization to anti-cancer drugs is attracting significant attention. Flavones, including apigenin, modulate AS on a gene-by-gene basis [10, 37–39]. However, the effects of natural compounds on reprogramming transcriptomes remain poorly understood. Here, we showed that the dietary nutraceutical apigenin induces global reprogramming of TNBC-associated AS, increasing isoforms associated with cell death. These effects were not observed in non-tumor breast epithelial cells, indicating the specificity of apigenin in switching ASI in TNBC (Fig. 4). Of significant physiological relevance, our results show that apigenin reprograms AS in vivo and reduces tumor growth (Fig. 6).

We previously showed that apigenin associates with RBPs, such as hnRNPA2, MSI2, and CELF1 [10]. Our results show that apigenin preferentially modulates the AS of hnRNPA2, MSI2, and CELF1 substrates (Fig. 3A). This is consistent with our findings that apigenin directly interacts with these RBPs as revealed in the screening of the human BC phage display peptide library (PD-Seq) [10]. In agreement with our findings, hnRNPA2, but not MSI2 and CELF1, was found as an apigenin-interacting protein in USO2 cells [37]. Additionally, apigenin interacts with splicing factor 3 C subunit B1 (SF3B1), a core subunit of the spliceosome, in USO2 cells [37]. These findings suggest that RBPs may exert cell type-specific activities. Together, the ability of apigenin to associate with hnRNPs and core spliceosome subunits suggests its potential to induce widespread AS modulation. Interestingly, the ΔPSI magnitude was significantly different only in hnRNPA2 substrates (Fig. 3B–D). Pathway analyses of the hnRNPA2 substrates affected by apigenin through AS revealed that the majority of the changes correspond to molecules involved in cell death and survival (Fig. 3E). Future studies assessing the contributions of these RBPs are warranted to further dissect how apigenin modulates cancer-specific AS circuitry and proteome. Notably, other flavones, including luteolin, biapigenin, hinokiflavone, and biflavone isoginkgetin, have been recognized as spliceosome inhibitors [40–42]. Altogether, these results support the ability of flavones to modulate AS and provide potential therapeutic options for TNBC treatment.

Our analysis of TNBC patient-derived RNA-seq data showed a significant increase in AS compared to non-tumor tissues (Fig. 5). Our results are consistent with other studies showing that a large fraction of AS events is dysregulated in BC [30, 43]. Our analyses revealed the ability of apigenin to reprogram the aberrant ASI found in the transcriptomes of TNBC patients to those found in non-tumor breast tissues (Fig. 5). RI was the most significantly enriched mRNA processing pattern affected by apigenin (Fig. 2E). In agreement with previous studies, we observed that the retention of introns was significantly enriched compared to intron exclusion in TNBC (Fig. 5B, C) [30], highlighting the relevance of this category in the pathogenesis of TNBC. Paradoxically, although intron retention is pervasively observed in most cancers, breast cancer usually displays low levels of intron retention compared to other cancers (Fig. 5B) [33]. Nevertheless, we observed that apigenin significantly targeted aberrant RI events in TNBC (Fig. 5F). RI typically results in the introduction of premature stop codons that trigger nonsense-mediated mRNA decay (NMD), inhibiting the protein synthesis of the affected mRNA [34, 44]. Alternatively, RI induces the expression of truncated peptides or proteins with extra domains, which can alter the function of canonical proteins or trigger protein turnover [34, 44].

We also showed that apigenin reprograms TNBC-dysregulated AS in vivo (Fig. 6), which was not observed in non-tumor breast epithelial cells (Fig. 4). Given that apigenin induces substantial death in TNBC cells but not in non-tumor breast epithelial cells (Fig. 1), we propose that reprogramming offers unique opportunities to overcome resistance. Supporting this hypothesis, we recently reported that apigenin sensitizes TNBC spheroids to doxorubicin-induced apoptosis [7]. Moreover, apigenin specifically sensitizes primary human lung epithelial cells isolated from human adenocarcinomas to TRAIL-induced apoptosis, without affecting matched non-tumor lung epithelial cells [9]. Additionally, apigenin is a sensitizer to immuno-therapies [45–47]. These results are impactful considering that aberrant cancer-associated AS affects 65% of the changes in the proteome [14], providing a rationale for the potential clinical relevance of apigenin in TNBC. Future studies comparing genome-wide AS perturbations triggered by nutraceuticals such as apigenin and anti-cancer compounds may reveal mechanisms offering opportunities for combinational therapies.

In conclusion, our findings reveal the impact of apigenin on the transcriptome of TNBC, providing novel mechanistic insights into the anti-proliferative and pro-apoptotic activities of this nutraceutical through transcriptome reprogramming. These findings suggest that transcriptome reprogramming triggered by apigenin may result in therapeutic vulnerability of cancer cells, which could present targeted therapeutic opportunities for TNBC.

### Supplementary information

Supplementary Material Methods

Fig. Supplementary 1

Fig. Supplementary 2

Fig. Supplementary 3

Supplementary Table 1

Supplementary Table 2

Supplementary Table 3

Supplementary Table 4

Supplementary Table 5

Supplementary Table 6

Supplementary Table 7

## Data Availability

The data generated in this study are publicly available in the GEO repository under the accession number GSE242297 and its Supplementary data files.

## Code availability

R packages and scripts used to analyze the data presented in this manuscript will be shared upon request.
