# Supplementary material for: Transcriptome reprogramming through alternative splicing triggered by apigenin drives cell death in triple-negative breast cancer
Source: Cell Death Dis. 2023 Dec 13;14(12):824. doi: 10.1038/s41419-023-06342-6 (PMC10719380; doi:10.1038/s41419-023-06342-6)
Supplement: Supplementary file 1 — Supplementary Material Methods [file 41419_2023_6342_MOESM1_ESM.docx]

**Supplementary Methods**

**Cell cycle analyses and proliferation**

Cell viability was determined by MTS assays using the CellTiter 96 Aqueous One Solution (Promega, Madison, Cat. #: G3582) using 6,000 cells and following the manufacturer’s instructions. For cell cycle analyses, cells were trypsinized, centrifuged, rinsed with PBS, and fixed in 70% ethanol. Fixed cells were rinsed twice with PBS and stained with 50 µg/ml propidium iodide (PI, Sigma-Aldrich, Cat. #: P4864) containing 0.2 mg/ml DNAse-free RNAse (Roche, Indianapolis, Cat. #: 11119915001) for 30 min at room temperature (RT). Cells were then analyzed by FACS using the BD Cell Quest Pro software (BD biosciences, San Jose, CA).

**Caspase-3 activity and apoptosis assays**

Caspase-3 activity was determined in cell lysates by DEVD-AFC assays (EMD Millipore, Cat. # 264150) as previously described^5^. Cell death was assessed by staining 60 cells/μl with 1 μg/ml calcein AM (ThermoFisher Scientific, Cat. #: C1430), as previously described^5^. The percentage of apoptotic cells was calculated as the number of cells stained with PI (red) plus cells stained with calcein AM and PI (green+red) divided by the total number of cells.

**Immunohistochemistry and TUNEL assays**

Proliferation in tissue sections was assessed by immunohistochemistry (IHC). Briefly, tumors were fixed in 10% buffered formalin, paraffin-embedded, longitudinally sectioned, and mounted onto glass slides. Tissue slides were deparaffinized in xylene and rehydrated in a series of 100%/95%/70% ethanol solutions before staining followed by antigen retrieval with 10 mM citrate buffer, pH 6 for 20 min at 125^o^C in a pressure cooker. Slides were washed twice with dH_2_O, incubated with 3% H_2_O_2_ in PBS for 15 min, and incubated in blocking solution containing 2% isotype control serum, 0.1% Tween-20 and 1% BSA in PBS for 1 h at RT. Slides were stained with mouse rabbit anti-Ki67 (Clone SP6 1:200, ThermoFisher Scientific, Cat. **#: MA5-14520, RRID: AB_10979488**) for 2 h at RT in a humidified chamber, rinsed 3 times in PBS followed by incubation with secondary biotin-conjugated antibodies (1:250, Vector Laboratories, provided with the Kit, Cat. #: PK-4002) for 30 min at RT and developed using the Vectastain ABC kit according to manufacturer’s instructions (Vector Laboratories, Cat. #: PK-4002). Slides were counter-stained with Hematoxylin (BBC Biochemicals), visualized under the light microscope, and blind counted using ImageJ. Ki67 index was calculated as the number of cells stained with Ki67 divided by the total number of cells in 25 random fields at 100X. Apoptosis was evaluated in tissue sections using the Terminal Uridine Nick-End Labeling (TUNEL) according to the manufacturer’s instructions (EMD Millipore, Cat. #: S7100). TUNEL-positive cells were scored in 25 random fields at 40X in a blinded manner and represented as counts per field (cpf).

**RNA extraction**

Total RNA was isolated from three independent biological replicates of MDA-MB-231 cells treated with 50 μM apigenin or diluent DMSO for 48 h by the Trizol method according to the manufacturer’s suggestions (ThermoFisher Scientific, Cat. #: 15596026), followed by 1U DNAse-I treatment (ThermoFisher Scientific, Cat. #: 18047019). For RNAseq analyses, RNA quality was evaluated by the Agilent Bioanalyzer 2100 (Agilent Technologies, Santa Clara, CA) and samples with RNA integrity number higher than 8.5 were used.

**AS analyses by isoform-specific RT-PCR**

Isolated RNA was reverse transcribed to cDNA and analyzed by RT-PCR and agarose gel electrophoresis as previously described^10^. The primers used are listed in Supplementary Table S3. PCR products were resolved on 1% agarose gels, bands were quantified by densitometry, and ΔPSI was calculated as 100*(Density of isoform X)/ (Density of the sum of all different isoforms).

**Enrichment of molecular functions**

The molecular and cellular functional enrichment analysis was performed using Ingenuity Pathway Analysis (IPA, QIAGEN Inc., [Ingenuity Pathway Analysis | QIAGEN Digital Insights](https://digitalinsights.qiagen.com/products-overview/discovery-insights-portfolio/analysis-and-visualization/qiagen-ipa/)). Statistical analyses were performed with the Benjamini-Hochberg correction testing with a *p*-value threshold of 0.05.

**Supplementary Figures**

**Supplementary Fig. S1**. **RNA-seq quality controls. A.** Description of reads and alignments. **B.** Hierarchical clustering and pairwise distances between the apigenin and DMSO-treated samples. **C.** Scatterplot matrix shows the distribution and correlation between samples and replicates.

**Supplementary Fig. S2. Validation of AS events affected by apigenin in MDA-MB-231 cells.** **A.** The AS events were segregated as excluded (blue) or included (yellow) in apigenin (Api) as compared to DMSO. Statistical significance was evaluated by Chi-square test with Bonferroni correction. **B.** Volcano plot of isoform-centric events depicts the percentage of spliced isoform (PSI) and the Log_2_ of the Bayesian factor. Changes with Bayes factor ≥ 20 are color-coded. **C.** AS events were validated by RT-PCR and gel electrophoresis. Data represent mean ± SEM, n=3. Statistical significance was evaluated by Two-tail Students’ *t*-test. **D.** Correlation of the ΔPSI values determined by RNA-seq *vs.* the ΔPSI values obtained by RT-PCR.

**Supplementary Fig. S3. Evaluation of AS events affected by apigenin in TNBC cell lines.**

**A.** AS events in mesenchymal BT-549 and basal-like MDA-MB-468 TNBC cell lines were evaluated by RT-PCR and gel electrophoresis. Data represent mean ± SEM, n=3. Statistical significance was evaluated by Two-tail Students’ *t*-test.

**Supplementary Tables**

**Supplementary Table 1.** List of AS events significantly affected by apigenin in MDA-MB-231 cells.

**Supplementary Table 2.** List of mRNA isoforms significantly affected by apigenin in MDA-MB-231 cells.

**Supplementary Table 3.** List of primers used for isoform-specific RT-PCR.

**Supplementary Table 4.** List of RBP substrates affected by apigenin.

**Supplementary Table 5.** List of cancer drivers, oncogenes, and tumor suppressors.

**Supplementary Table 6.** List of AS events involved in cell death and survival affected by apigenin.

**Supplementary Table 7.** List of AS events significantly dysregulated in human TNBC patients.
