# Supplementary material for: Transcriptome reprogramming through alternative splicing triggered by apigenin drives cell death in triple-negative breast cancer
Source: Cell Death Dis. 2023 Dec 13;14(12):824. doi: 10.1038/s41419-023-06342-6 (PMC10719380; doi:10.1038/s41419-023-06342-6)

Supplementary Fig. 1

A

Sample Treatment	Total Reads	Total Aligned Reads	% Alignment
DMSO-1	48,066,452	43,256,467	98.75%
DMSO-2	45,786,382	40,974,335	98.70%
DMSO-3	48,116,463	41,456,058	98.59%
Apigenin-1	39,253,608	34,334,866	98.15%
Apigenin-2	40,472,211	35,583,602	98.26%
Apigenin-3	48,130,199	41,542,356	98.16%

B

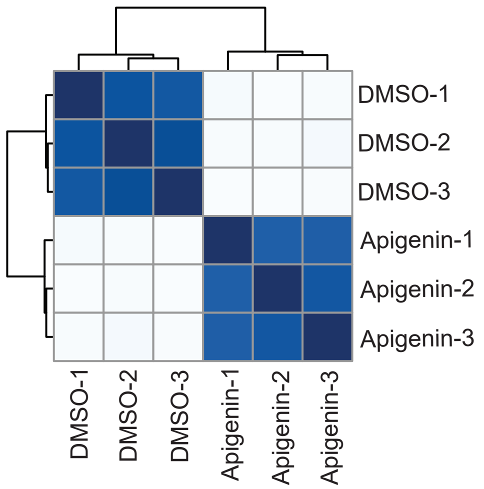

C

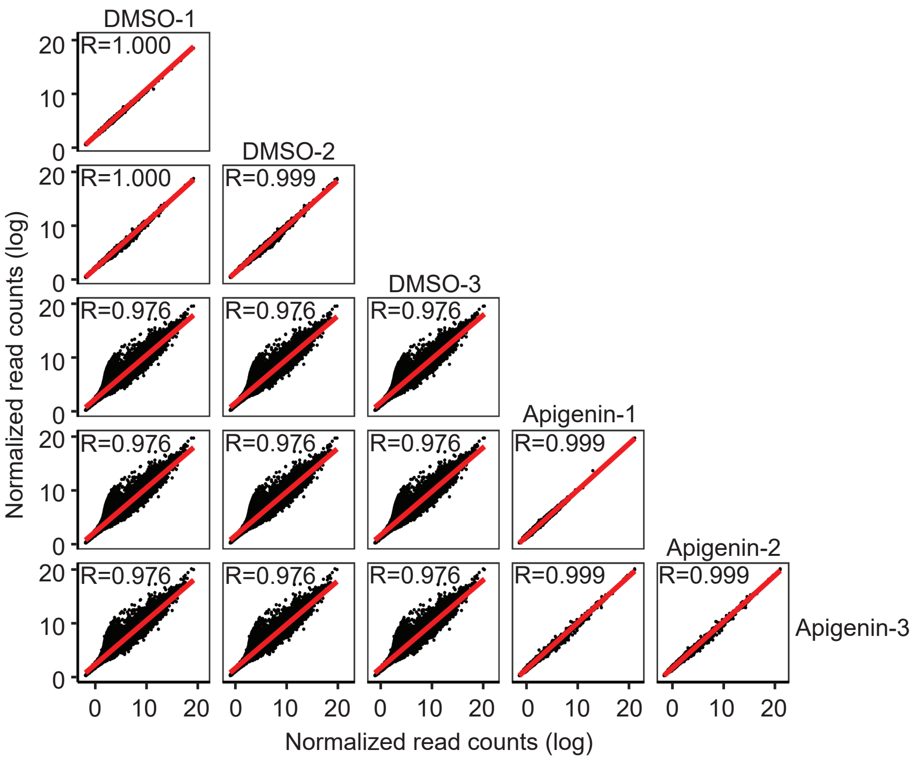

Supplement: Supplementary file 2 — Fig. Supplementary 1 [file 41419_2023_6342_MOESM2_ESM.pdf]
