# Supplementary figures and images for: Transcriptome reprogramming through alternative splicing triggered by apigenin drives cell death in triple-negative breast cancer
Source: Cell Death Dis. 2023 Dec 13;14(12):824. doi: 10.1038/s41419-023-06342-6 (PMC10719380; doi:10.1038/s41419-023-06342-6)

Supplementary Fig. 2

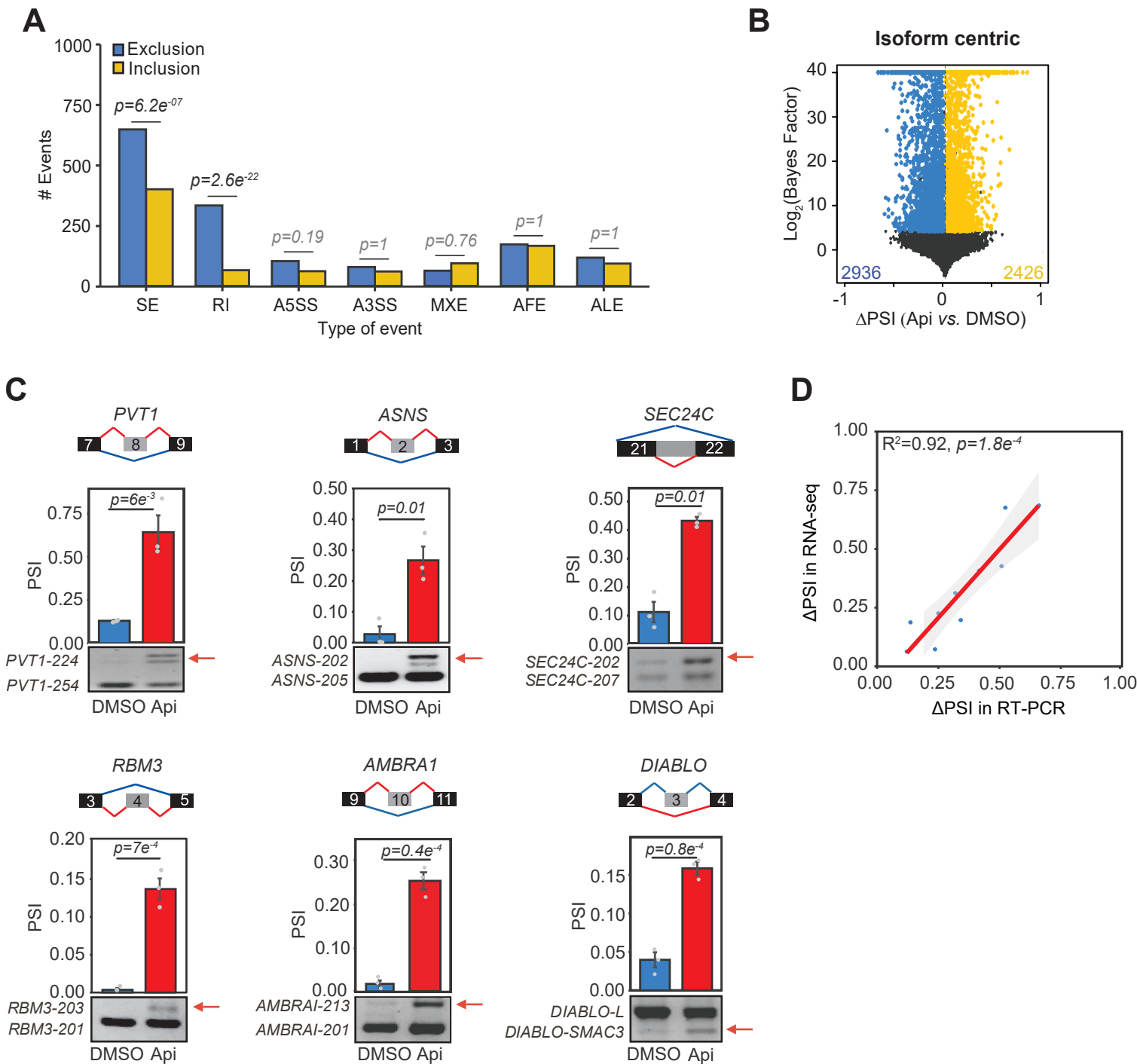

Supplement: Supplementary file 3 — Fig. Supplementary 2 [file 41419_2023_6342_MOESM3_ESM.pdf]

# Supplementary Fig. 3

A

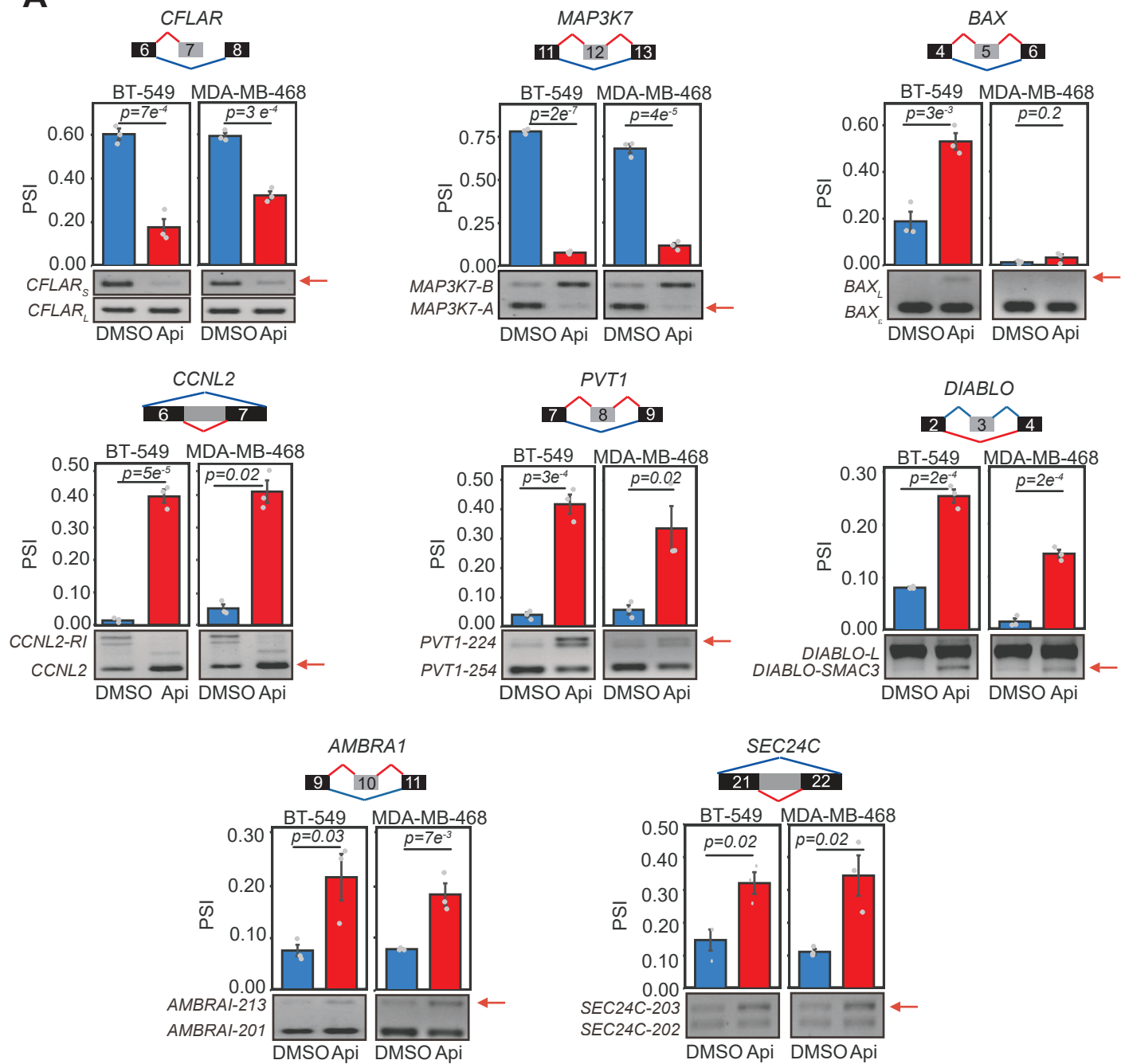

Supplement: Supplementary file 4 — Fig. Supplementary 3 [file 41419_2023_6342_MOESM4_ESM.pdf]
